# A novel strain of *Leishmania braziliensis* harbors not a toti- but a bunyavirus

**DOI:** 10.1371/journal.pntd.0012767

**Published:** 2024-12-27

**Authors:** Alexei Yu. Kostygov, Danyil Grybchuk, Senne Heeren, Evgeny S. Gerasimov, Donnamae Klocek, Aditya Reddy, Jovana Sádlová, Lenka Pacáková, Alain Kohl, František Stejskal, Petr Volf, Jean-Claude Dujardin, Vyacheslav Yurchenko

**Affiliations:** 1 Life Science Research Centre, Faculty of Science, University of Ostrava, Ostrava, Czechia; 2 Zoological Institute of the Russian Academy of Sciences, St. Petersburg, Russia; 3 Central European Institute of Technology, Masaryk University, Brno, Czechia; 4 Department of Biomedical Sciences, Institute of Tropical Medicine, Antwerp, Belgium; 5 Department of Microbiology, Immunology and Transplantation, Rega Institute for Medical Research, Katholieke Universiteit Leuven, Leuven, Belgium; 6 Department of Biomedical Sciences, University of Antwerp, Antwerp, Belgium; 7 Faculty of Biology, Lomonosov Moscow State University, Moscow, Russia; 8 Department of Parasitology, Faculty of Science, Charles University, Prague, Czechia; 9 Centre for Neglected Tropical Diseases, Departments of Tropical Disease Biology and Vector Biology, Liverpool School of Tropical Medicine, Liverpool, United Kingdom; 10 Department of Infectious Diseases, 2^nd^ Faculty of Medicine and Clinics of Infectious, Parasitic, and Tropical Diseases, Bulovka University Hospital, Charles University, Prague, Czechia; 11 Department of Infectious Diseases, Regional Hospital Liberec, Liberec, Czechia; Centro de Pesquisa Gonçalo Moniz-FIOCRUZ/BA, BRAZIL

## Abstract

*Leishmania* is a genus of the family Trypanosomatidae that unites obligatory parasitic flagellates causing a variety of vector-borne diseases collectively called leishmaniasis. The symptoms range from relatively innocuous skin lesions to complete failures of visceral organs. The disease is exacerbated if a parasite harbors *Leishmania* RNA viruses (LRVs) of the family *Pseudototiviridae*. Screening a novel isolate of *L*. *braziliensis*, we revealed that it possesses not a toti-, but a bunyavirus of the family *Leishbuviridae*. To the best of our knowledge, this is a very first discovery of a bunyavirus infecting a representative of the *Leishmania* subgenus *Viannia*. We suggest that these viruses may serve as potential factors of virulence in American leishmaniasis and encourage researchers to test leishmanial strains for the presence of not only LRVs, but also other RNA viruses.

## Author summary

Parasites of the genus *Leishmania* cause a series of neglected tropical diseases collectively known as leishmaniasis. Many isolates of these parasites possess *Leishmania* RNA viruses (LRVs) of the family *Pseudototiviridae* that increase a chance of developing more severe mucocutaneous leishmaniasis over the cutaneous form, facilitate the spread of leishamaniae, and make these flagellates more resistant to treatments. In this work, we demonstrate that pseudototiviruses are not the only infecting agents and some isolates of *Leishmania* may harbor other viruses, exemplified by the leishbuvirus *Lbr*LBV1 of *L*. *braziliensis*. Because they may also be considered as potential factors of virulence, we advocate for routine testing of leishmanial strains for the presence of not only LRVs, but also other RNA viruses.

## Introduction

Trypanosomatids (Euglenozoa: Kinetoplastea: Trypanosomatidae) are a group of flagellates, whose members represent obligate parasites of invertebrates, plants, and vertebrates [1,2]. They have either one (monoxenous species) or two (dixenous species) hosts in their life cycles [3,4]. Many dixenous trypanosomatids are medically and/or economically important, yet they all can be traced back to their inconspicuous and unharmful (from the anthropocentric point of view) monoxenous kin, from which they have independently originated at least thrice in evolution [5]. One of such transitions to dixeny happened within the subfamily Leishmaniinae, facilitating emergence of the well-known genus *Leishmania* [6,7]. These flagellates, transmitted mostly by phlebotomine sand flies, infect vertebrates and cause a variety of diseases collectively named leishmaniases, which range from relatively innocuous skin lesions to complete failures of visceral organs [8]. The genus *Leishmania* is subdivided into four subgenera–*Leishmania*, *Mundinia*, *Sauroleishmania*, and *Viannia*, which can be defined phylogenetically and by details of their respective life cycles [9]. Genomes of numerous *Leishmania* spp. have been sequenced and scrutinized both bioinformatically and functionally [10–14].

Many trypanosomatids are known to harbor RNA viruses [15,16]. The very first virus-like particles in these flagellates were described a half-century ago in *Porcisia hertigi* (at that time classified as a member of the genus *Leishmania*) [17]. The pioneering molecular works were performed on leishmaniaviruses (double-stranded RNA viruses of the family *Pseudototiviridae*, order *Ghabrivirales* [18]) infecting *L*. (*Viannia*) and *L*. (*Leishmania*) spp. in the New and Old worlds, respectively [19–21]. In the case of LRV-1 (*Leishmaniavirus ichi*), it has been convincingly demonstrated that its presence in *L*. (*V*.) *guyanensis* and *L*. (*V*.) *braziliensis* is linked to the augmented parasite burden and immune response *in vitro* [22–24], as well as the severity of leishmaniasis and drug-treatment failures in patients [25–27]. LRV-2 appears to be restricted to *L*. (*Leishmania*) and *L*. (*Sauroleishmania*) spp. [28–32] and its effects on parasite biology might differ from those elicited by LRV-1 [33–35].

To the best of our knowledge, no viruses other than LRVs have been documented in *L*. (*Viannia*) and *L*. (*Leishmania*) spp. The situation is different in two other *Leishmania* subgenera–*Mundinia* and *Sauroleishmania*. Out of the four screened isolates of *L*. (*Mundinia*) one was shown to possess a leishbuvirus [36], while a narnavirus and a novel lineage of LRV-2 were documented in three out of seven isolates of *L*. (*Sauroleishmania*) analyzed [32].

Leishbuviruses (*Negarnaviricota*: *Polyploviricotina*: *Bunyaviricetes*: *Leishbuviridae*) [37] appear scarce in *Leishmania* but fairly prevalent in monoxenous trypanosomatids of the genera *Blechomonas*, *Crithidia*, and *Leptomonas* [16,38–41]. The family currently contains a single genus *Shilevirus* [42] and its members are exclusively associated with trypanosomatids. This suggests that their ancestors switched from insects (the predominant hosts in the outgroup) to trypanosomatids only once, after which horizontal transfer events determined co-speciation of these viruses with their new hosts. Similarly to other viruses of the class *Bunyaviricetes*, leishbuviruses have a tripartite genome with large (L), medium (M), and small (S) segments bearing terminal complementary sequences that facilitate replication [43] and encoding an RNA-dependent RNA polymerase (RdRp), a surface glycoprotein, and a nucleocapsid protein, respectively [44]. These viruses form enveloped virions 90–100 nm in diameter, where viral glycoproteins are incorporated into the membrane envelope [45,46].

In this work, we demonstrate that in addition to LRVs, *Leishmania* (*Viannia*) *braziliensis* can be infected by bunyaviruses. The *Lbr*LBV1 virus identified in this work differs from other described leishbuviruses in sequences of its terminal repeats. The *L*. *braziliensis* cells without *Lbr*LBV1 behave similarly to their virus-positive counterparts in terms of the cell division kinetics and development in the sand flies. Yet, the complex biological consequences of possessing a bunyavirus by *Leishmania* spp. need to be investigated further. We suggest that these viruses may serve as potential factors of virulence in American leishmaniasis and encourage investigators and practitioners to test leishmanial strains for the presence of not only LRVs, but also other RNA viruses.

## Materials and methods

### Ethics statement

Collection of the *Leishmania* sample was approved by the Ethics Committee of the Clinics of Infectious, Parasitic, and Tropical Diseases, Bulovka University Hospital under the approval number 9214/EK-Z. A formal verbal consent was obtained from the patient.

### Clinical history, strain isolation and cultivation, genomic DNA and total RNA isolation, species validation, and analysis of viral presence

**Fig 1:**
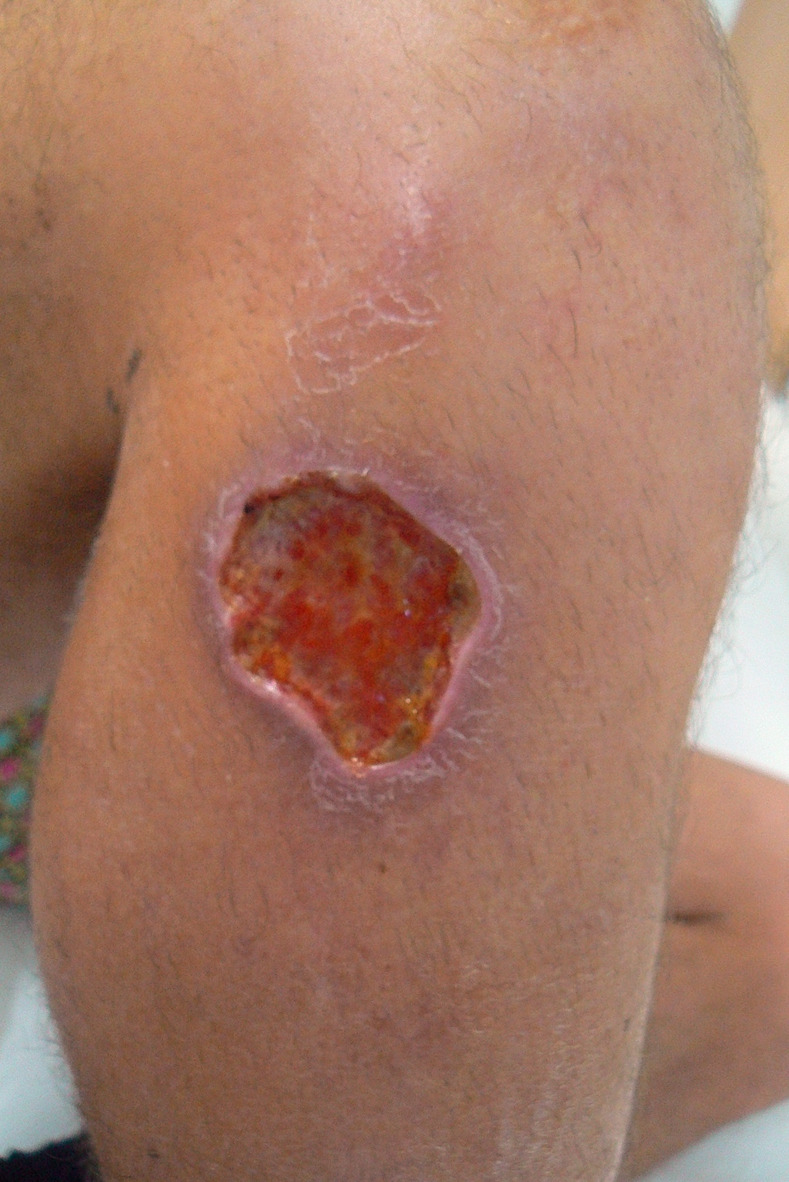
Clinical manifestation of cutaneous leishmaniasis caused by *L*. *braziliensis* BO17.

A healthy 31-year-old male Czech tourist visited Argentina, Chile, Bolivia, Ecuador, Peru, and Colombia from November 2016 to August 2017. The traveler reported numerous sand fly bites in Rurrenabaque, a popular tourist destination in the north-western Bolivia (14°26’ S, 67°31 W), in March 2017. Around mid-July, 2017, a boil with a crust appeared on the right shank just under the knee (Fig 1) and was painless on the onset. Its size increased from 1 to 2.5 cm (both values are approximate) over a few weeks, accompanied by the sanguinolent discharge. On July 27^th^, 2017, the patient was seen at the local clinics in Colombia and treated with the antibiotics ceflexin (p.o.) and sulfadiazine (topical) with no effect. Because a secondary bacterial infection has developed, the patient was admitted to the same local hospital and treated with oxacillin and clindamycin (both i.v.). He was later transferred to the larger Hospital Universitario San Vicente Fundación in Medellin, Colombia, where the treatment with clindamycin (p.o.) continued for 3 days. The biopsy from the lesion tested positive for leishmaniasis, and the thermotherapy [47] was performed on August 3^rd^, 2017. The patient was prescribed Alyeyuba cream and lotion (both topical) for 28 days. The patient returned to the Czech Republic on August 14, 2017 and the second biopsy taken in Prague on August 23^rd^ confirmed *Leishmania* sp. infection. The blood test results were largely inconspicuous: white blood cell count and differential count were normal, biochemistry was normal, CRP was 3.9 mg/l, and alanine aminotransferase level was slightly elevated at 104 units/l. The serology for leishmaniasis was negative. The patient refused the recommended treatment with antimony and, instead, continued with Alyeyuba application. Over the next two months, the lesion doubled in size, which was accompanied by the enlargement of inguinal lymph nodes. A treatment with the antimony drug Glucantime (20 mg/day (i.m.)) lasted from October 19^th^ till November 8^th^, 2017, followed by the intralesional application of 2 ml Glucantime (1.5 g/5 ml) once a week for 3 weeks. As there was discharge from the two smaller lesions under the knee, the treatment with itraconazole was continued under the following regimen: 400 mg daily from December 14^th^ to December 27^th^, 2017; 300 mg daily from December 28^th^, 2017 to January 7^th^, 2018; 200 mg daily from January 7^th^ to January 14^th^, 2018, when the treatment was discontinued due to an allergic reaction. The secondary bacterial infection in the scar was treated with clarithromycin in April 2024. There was no relapse of the leishmanial infection.

The parasite strain isolated from this patient was designated as *Leishmania braziliensis* MHOM/BO/17/BO17 (hereafter referred to as BO17 for short). The flagellates were cultivated in the M199 medium (Sigma-Aldrich/ Merck, Darmstadt, Germany) supplemented with 2 μg/ml Biopterin (Merck), 2 μg/ml Hemin (Jena Bioscience GmbH, Jena, Germany), 25 mM HEPES (VWR/ Avantor, Radnor, USA), 50 units/ml of penicillin, 50 μg/ml of streptomycin (both from Biowest, Nuaillé, France), and 10% fetal bovine serum (Biosera, Cholet, France) at 25°C as described previously [48].

Total genomic DNA and RNA were isolated from 10 ml of trypanosomatid cultures with the DNeasy Blood & Tissue and RNeasy Mini kits (both from Qiagen, Hilden, Germany) according to the manufacturer’s instructions. The cultured species affinity to the subgenus *Viannia* was initially confirmed by 18S rRNA gene amplification and sequencing as described previously [49]. The detection of viral dsRNA in the studied isolate was performed according to a previously described method [50]. The strains *L*. (*V*.) *guyanensis* MHOM/BR75/M4147 (bearing LRV-1) and *L*. (*S*.) *hoogstraali* RHEM/SD/1963/NG-26 (virus-free) were used as positive and negative controls, respectively [10,32].

### Whole-genome and transcriptome sequencing, assembly and variant calling

DNA and RNA libraries were prepared as described previously [51] and sequenced on NovaSeq X (Illumina, San Diego, USA) at Macrogen Europe (Amsterdam, Netherland) in paired-end mode with a read length of 150 bp. The sequencing runs produced 36.7 million and 4.9 million reads for DNA and RNA libraries, respectively, with the average base Q score of 36. The obtained raw sequencing data were deposited in GenBank (BioProject PRJNA1086002).

Genomic reads were quality-checked with FastQC v. 0.12.1 [52] and trimmed with fastp v. 0.23.4 [53]. These reads and those for a number of *L*. (*Viannia*) spp. isolates (S1 Table) were mapped to *L*. *braziliensis* MHOM/BR/75/M2904 2019 genome assembly (the most recent reference sequence for this species available on the TriTrypDB [54]) using SMALT v.0.7.6 [55] with the following parameters: k = 13; s = 2. Variant calling was performed using the Genome Analysis Toolkit GATK v. 4.1.4.1. [56] in several steps: i) calling of SNPs and indels in each sample with HaplotypeCaller; ii) uniting individual gVCF files with CombineGVCFs; iii) joint genotyping of samples with GenotypeGVCFs; iv) separation of SNPs from INDELs with SelectVariants; and v) filtration of SNP calls with VariantFiltration using the following criteria: QD < 2; FS > 60.0; MQ < 40.0; SOR > 3.0; MQRankSum < -12.5; ReadPosRankSum < -8.0; QUAL < 100; Format DP < 5; Format GQ < 30.

Raw RNA reads were trimmed using Trimmomatic v. 0.40 [57] and assembled *de novo* with Trinity v. 2.13.2 [58]. To estimate coverage, reads were mapped back to the assembled contigs using Bowtie 2 v. 2.4.4 [59] and sorted with SAMtools v. 1.13 [60]. Per-base coverage was calculated using BEDTools v. 2.30.0 [61] and, based on that, per-contig RPKM (Reads Per Kilobase per Million) values were computed with a custom *awk* script.

### Phylogenetic analyses and assessment of potential hybrid ancestry of the studied isolate

A phylogenetic network was reconstructed using SplitsTree v. 4.17 with default parameters [62] based on 1,055,633 genome-wide biallelic SNPs that were inferred for the BO17 and 49 additional isolates (S1 Table). The phylogeny reconstruction based on 92 maxicircle SNPs identified as described previously [63] for the same set of isolates, was performed by the maximum likelihood method in IQ-TREE v. 2.3.4 with substitution model TN + F + ASC as chosen by the built-in ModelFinder and branch support assessed using 100 standard bootstrap replicates [64].

Species-level ancestry of the BO17 isolate was assessed with PCAdmix v. 1.0 [65] based on the Beagle v. 5.2 [66] phased genotype data (433,086 SNPs) for the dataset including 28 isolates of *L*. *braziliensis*, *L*. *peruviana*, and their hybrids (S2 Table). Local ancestry was inferred across the genome as described previously in bins of 20 SNPs using three isolates per each of the following categories: *L*. *braziliensis* from Peru and Bolivia (parent), *L*. *peruviana* from Peru (parent), and known *L*. *braziliensis* × *L*. *peruviana* hybrids [67]. Potential intraspecific hybrid ancestry of the studied isolate was investigated as above using a dataset of 169,519 SNPs from the previous study [67] including three populations of *L*. *braziliensis* L1 from Peru and Bolivia (PAU, INP, and HUP) as putative ancestral/parental groups and one hybrid population (ADM).

### Identification of viral sequences and phylogenetic analysis of viruses

Leishbuviral segments L and S were detected using DIAMOND v. 2.0.2 [68] search of all assembled contigs against Uniclust50 protein database [69]. Less conserved segment M was found by TBLASTn search of the *Leishmania martiniquensis leishbuvirus 1* glycoprotein sequence [36,70] against a nucleotide database of assembled contigs. Open reading frames were annotated with NCBI’s ORF Finder web tool [71]. Terminal complementary sequences were identified and visualized using IPknot v. 2.2.1 [72].

Phylogeny of the discovered leishbuvirus was inferred from the amino acid sequence of segment L gene product—a multifunctional protein with RdRp activity [73]. The dataset included previously reported *Leishbuviridae* and *Phenuiviridae* (as an outgroup). Sequences were aligned using G-INS-i algorithm in MAFFT v. 7.490 [74] with a maximum of 1,000 iterations. A series of trimmed alignments with different gap thresholds (from 0.2 to 0.975 in steps of 0.025) was produced with trimAl v. 1.4 [75]. Each alignment was used to test the substitution model and build a phylogenetic tree with ultra-fast bootstrap supports using IQ-Tree v. 2.2.5. One alignment with the highest average ultra-fast bootstrap value (gap threshold 0.8) was selected for phylogenetic inference in IQ-Tree with 1,000 standard bootstrap replicates and the automatically selected best-fit model LG + I + F + G4. The same alignment and model were used for Bayesian inference in MrBayes v. 3.2.7. [76]. All other settings were left in their default states.

### Obtaining the virus-free clonal lines and assessing growth curves

The BO17 culture was passaged every 10–12 days for two months allowing the culture to reach the post-plateau stage when the concentration of cells started to decline prior to sub-culturing. The culture was then spread onto 1% agar/supplemented M199 medium plates as described previously [77], and total RNA was extracted from 15 clonal colonies. Complementary DNA was synthesized from 1 μg of total RNA from each clone using Transcriptor First Strand cDNA Synthesis Kit (Roche Diagnostics, Indianapolis, USA) following the manufacturer’s instructions. Abundance of LBV was estimated by RT-qPCR using the primers LBV_f: ttcattgccaccagatttgccc and LBV_r: acatcacccaataccgattccc and normalization to 60S ribosomal protein L7a [78]. Identities of the two virus-negative clones (named hereafter LBV(-) 1 and LBV(-) 2) were verified via sequencing of their genomic 18S ribosomal RNA locus as described previously [79].

For growth kinetics, the original BO17 culture (wild-type) and the two virus-negative lines were seeded at the density of 5 × 10^5^ parasites per ml in triplicates. Parasite concentrations were determined every 24 hours for 7 days as described previously [80].

### Infection of sand flies

Established laboratory colonies of the sand flies *Lutzomyia longipalpis* (from Jacobina, Brazil) and *Lu*. *migonei* (from Baturité, Brazil) were maintained under standard conditions [81]. *Lutzomyia longipalpis* is a frequent laboratory model permissive for several *Leishmania* spp., including *L*. *braziliensis* [82], while *Lu*. *migonei* is a natural vector of this parasite in eastern Brazil [83]. For sand flies’ infection, the wild type (WT) and LBV-negative cultures were maintained at 23°C in M199 (Sigma-Aldrich/ Merck) supplemented with 10% fetal calf serum (Thermo Fisher Scientific, Waltham, USA), 1× BME vitamins (Sigma-Aldrich/ Merck), 2% human urine, and 250 μg/ml amikin (Bristol-Myers Squibb, New York, USA).

Female sand flies (3–6 days old) were fed through a chick skin membrane on heat-inactivated ram blood (Bioveta International, Ivanovice na Hané, Czechia) containing 10^6^ promastigotes/ml. Engorged females were maintained in the same conditions as the colony and dissected on days 3 and 10 post bloodmeal (PBM). Individual guts were analyzed by light microscopy for localization and intensity of infection. Parasite loads were graded according to [84] as light (< 100 parasites per gut), moderate (100 to 1,000 parasites per gut), and heavy (> 1,000 parasites per gut). The results were summarized for two independent biological experiments, and differences between groups in infection rate and location of infection at the stomodeal valve were calculated by “N-1” χ^2^ test [85].

## Results

### Genome analysis and classification of the patient-derived *Leishmania* isolate

The genome of *L*. *braziliensis* MHOM/BO/17/BO17 was sequenced to the median coverage of 266×, with only 0.2% of positions in the reference genome completely uncovered.

**Fig 2:**
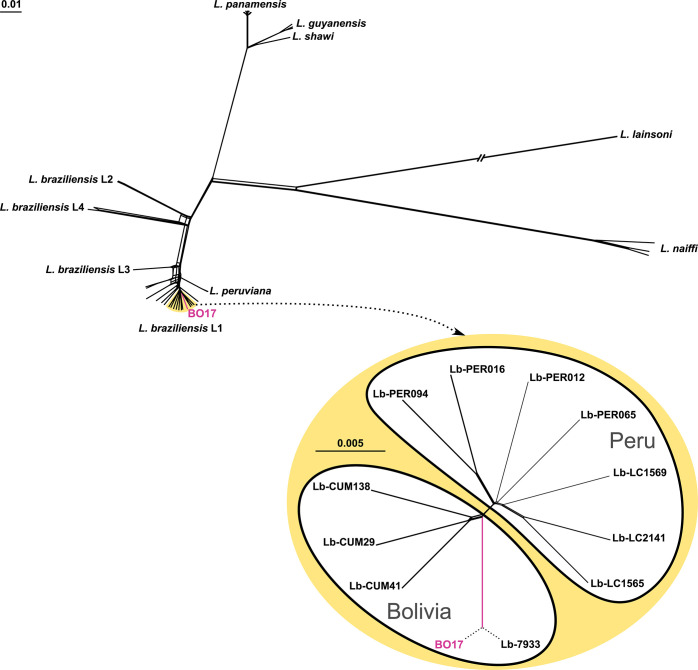
Phylogenetic networks of *L*. (*Viannia*) based on genome-wide SNPs. Upper panel depicts the relationships at the subgenus level and the lower panel provides the closer view on the clade (highlighted in yellow) containing the BO17 isolate that is shown in magenta. The double-crossed branch is at 50% of its length. The scale bar denotes the number of substitutions per site.

As *L*. *braziliensis* represents a phylogenetically complex taxon, which has close relationships with *L*. *peruviana* [86–88], we decided to perform a detailed phylogenetic analysis of the isolate under study. The phylogenetic network based on the nuclear SNPs convincingly demonstrated the position of the BO17 isolate within the lineage L1 of *L*. *braziliensis* [89] (Fig 2). A closer look at the clade under scrutiny revealed that BO17 clusters within a group of Bolivian isolates and shares the same branch with the isolate Lb-7933 [90], suggesting that their genomes are nearly identical. Indeed, analysis of SNPs in these two isolates identified only 21 heterozygous and 3 homozygous differences. Thus, BO17 is a Bolivian isolate of *L*. *braziliensis* belonging to the lineage L1. A similar situation was documented when phylogenetic relationships were inferred using kDNA data (S1 Fig).

Considering the fact that *L*. *braziliensis* can form hybrids with *L*. *peruviana* [86,91], we additionally assessed the ancestry of the studied isolate. The PCA plots produced using PCAdmix demonstrated that, while the ancestry of the parental samples matched well with their respective species identities, the *L*. *braziliensis* × *L*. *peruviana* hybrids occupied an intermediate position between the two parents. The BO17 isolate unambiguously clustered with *L*. *braziliensis* showing no signs of admixture of *L*. *peruviana* genome (S2 Fig.). However, our analysis at the intraspecific level demonstrated that all three previously characterized ancestral populations of *L*. *braziliensis* L1 lineage (HUP, PAU, and INP) contributed to the formation of the genomes of BO17 and its closest relative, Lb-7933. This was illustrated by the intermediate position in the PCA scatterplot and mosaic composition of chromosomes (S3 Fig). Thus, we concluded that the BO17 strain belongs to a hybrid population of *L*. *braziliensis* within the lineage L1 from Bolivia.

### Viral presence and virus sequence analysis

**Fig 3:**
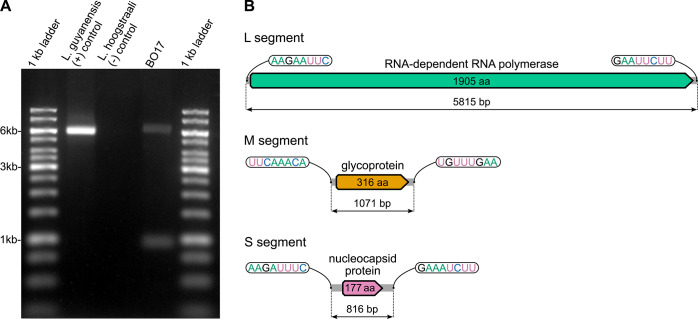
*Lbr*LBV1 of *L*. *braziliensis* BO17. (A) Agarose gel visualization of BO17 dsRNA. *Leishmania guyanensis* dsRNA was used as a positive control. GeneRuler 1kb DNA Ladder was added as size reference. (B) Representation of *Lbr*LBV1 genomic segments (drawn to scale). Directional shapes depict predicted ORFs with the lengths of encoded proteins displayed inside. Terminal repeats are shown in callouts.

The analysis of *L*. *braziliensis* BO17 dsRNA preparation allowed visualization of two bands of approximately 1 and 6 kb (Fig 3A). Of note, the mobility of dsRNA in the agarose gel differs from that of DNA, and the bands are shifted upwards. This pattern is consistent with the presence of a leishbuvirus (the M segment is often not detectable on a gel [40,41]). Notably, the abundance of dsRNA in this sample was lower than that of LRV-1 in *L*. *guyanensis* M4147 used as a positive control despite the same starting concentration of total RNA. This is not surprising since, in contrast to LRVs, which are genuine dsRNA viruses, LBVs have dsRNA only as a replicative intermediate.

Analysis of the BO17 transcriptome revealed a leishbuvirus (hereafter called *Lbr*LBV1 = *Leishmania braziliensis leishbuvirus 1*) with three genomic segments sizing 5.8, 1.1, and 0.8 kb, each encoding a single ORF (Fig 3B) The lengths of RNA segments and respective ORFs were typical for the “crown” clade of LBVs [38] (S3 Table). The relative abundance of the three RNAs estimated based on whole-transcriptome sequencing data was significantly different from their double-stranded variants observable on the gel. This could be explained by the different levels of strandedness bias and/or efficiency of dsRNA preservation during its preparation. All three segments contained terminal complementary sequences forming "panhandle" structures (Figs 3B and S4) characteristic for *Bunyaviricetes* [92]. Nevertheless, the sequences of these terminal repeats considerably differed from those in all other *Leishbuviridae* investigated so far: instead of the typical ACACAAAG, *Lbr*LBV1 termini were AAGA(A/U)UUC and UUCAAACA for the L/S, and M segments, respectively (Fig 3B). Non-canonical and different between segments terminal repeats have been previously documented only in more divergent LBVs of the firebug-infecting trypanosomatid *Leptomonas pyrrhocoris* [38].

**Fig 4:**
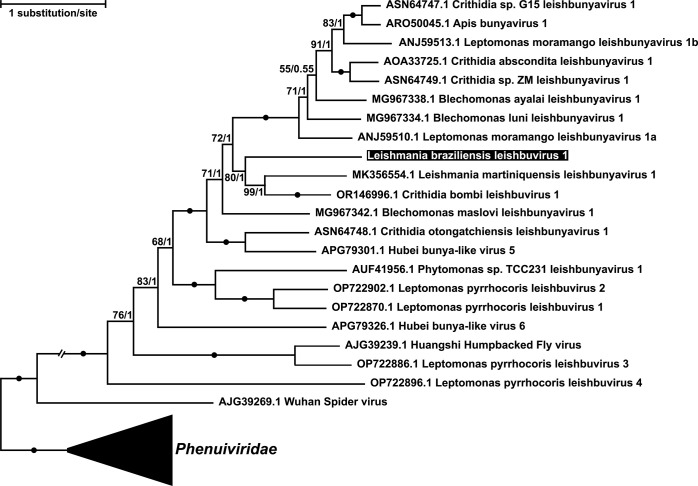
Phylogenetic tree of *Leishbuviridae* based on the RDRP amino acid sequences. The tree was rooted on *Phenuiviridae* (collapsed for better visibility). *Lbr*LBV1 is highlighted in black. Numbers at nodes are standard bootstrap supports/Bayesian posterior probabilities, circles indicate support of 100/1.

Phylogenetic analysis demonstrated a sister relationship of *Lbr*LBV1 with the clade encompassing *Lmar*LBV1 and *Cbom*LBV1. These three viruses were nested within a large cluster of viruses from *Leptomonas moramango* and various species of *Crithidia* and *Blechomonas* (Fig 4).

### Viral load during cultivation and comparison of virus-positive and -negative clones

**Fig 5:**
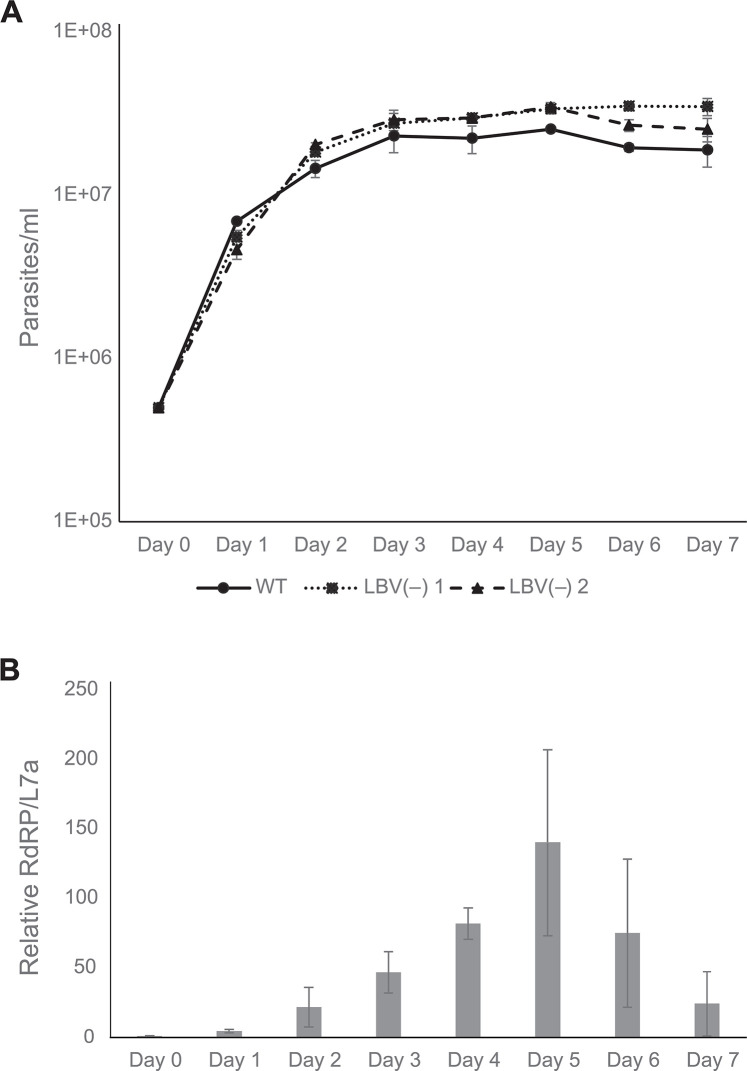
Growth curves and viral load of *L*. *braziliensis* BO17. (A) Growth curves of the WT, LBV(-) 1, and LBV(-) 2 cultures. Data are calculated from three independent biological replicates. (B) Viral load in the WT culture analyzed by quantitative RT–PCR of RdRP mRNA. Data are summarized from three independent biological replicates, each with three technical replicates. Error bars indicate standard deviation.

Next, we obtained two virus-negative clones: LBV(-) 1 and LBV(-) 2. The analysis of growth kinetics demonstrated that the WT strain proliferated a bit slower and reached about one third lower density as compared to the virus-free clonal strains (Fig 5A). Although being small, this difference was statistically significant (*p* < 0.005 at day 5 by t-test). This suggests that the presence of the virus may have a slight deleterious effect on the cells. We also noted that viral prevalence was not stable: it increased during the log and early plateau phases reaching maximum at day 5 after passaging and went down afterwards (Fig 5B).

Finally, we experimentally infected two *Lutzomyia* spp. (*Lu*. *longipalpis* and *Lu*. *migonei*) [83] with either the wild-type or one of the two virus-negative clonal strains of *L*. *braziliensis*. In *Lu*. *migonei*, the overall infection rates by all three strains were not significantly different (*p* = 0.1736 and 0.0504; χ2 = 1.851 and 3.830 for days 3 and 10 PBM, respectively). In *Lu*. *longipalpis*, the overall infection rates of LBV(-) 1 clone were about 20% lower than those of the wild-type on day 3 PBM (*p* = 0.0125; χ^2^ = 6.240) as well as on day 10 PBM (*p* = 0.0042; χ^2^ = 8.202), whereas LBV(-) 2 infection rates did not differ significantly from the WT (*p* = 0.0687 and 0.7486; χ^2^ = 3.313 and 0.103 for days 3 and 10 PBM, respectively). (S5 Fig). Considering that the statistically significant difference was observed only for one clone and one sand fly species, as well as the relatively small extent of this difference, we concluded that the virus did not essentially impact development of *L*. *braziliensis* in the vector.

## Discussion

In this work, we investigated a leishmanial strain isolated from a Czech patient who had travelled to South America and identified a novel virus in it. The analysis of genomic data for this strain allowed us unambiguously identifying not only the parasite species (*Leishmania braziliensis*) and lineage (L1), but even the geographic origin of the strain (Bolivia), about which the anamnesis data were inconclusive. Our inference excluded interspecific hybrid ancestry of the isolated parasite, which was important considering that *Leishmania* spp. tend to hybridize [93–99]. However, the strain under study appears to be a result of intraspecific hybridization, which has been recently demonstrated to be associated with elevated frequency of infections by leishmaniaviruses [67]. By the analogy, the same effect could possibly be exerted for LBVs, but a single currently available example is not enough for making any sound conclusion. Of note and in contrast to LRVs, LBVs form enveloped virions, which should significantly facilitate horizontal viral transmission between host species [16,36].

The evolutionary origin *Lbr*LBV1 cannot be unambiguously established, because the known diversity of the family *Leishbuviridae* is still relatively scarce. However, our phylogenetic inference indicates that it arose independently from the related virus of *L*. (*M*.) *martiniquensis* (*Lmar*LBV1) [36]. In both cases, the viruses were likely acquired from monoxenous trypanosomatids, which are known to co-habit vector’s intestine with *Leishmania* spp. [100–102]. Of note, the distinctness of vectors for *L*. *braziliensis* and *L*. *martiniquensis* (sandflies and biting midges, respectively) is in line with the independent origin of viruses in these flagellates [103].

The discovered virus belongs to the crown group of *Leishbuviridae* and resembles its relatives in the sizes of genomic segments and ORFs. However, it possesses terminal complementary sequences not only distinct from the canonical ones, but even not identical for all the genomic segments. This phenomenon, previously detected only in the divergent viruses from the monoxenous trypanosomatid *Leptomonas pyrrhocoris* [38], warrants special attention, since it is unclear what could be the functional consequences of such a discrepancy. It may, for example, impact replication and transcription of a given segment, suggesting that expression of specific gene products may require specific regulation. In addition, the uniqueness of the terminal sequences for the M segment suggests that it has a different evolutionary origin, i.e., *Lbr*LBV1 may be a reassortant.

Our observation that the viral load increases during the log and early plateau stages of the culture growth and subsequently decreases afterwards may have two explanations. Firstly, in some cells, the LBV proliferation may occur faster than in others. This would lead to subsequent elimination of such cells from the culture due to a presumable toxic effect. The persistence of viruses in the population can then be ensured by the cells, in which the multiplication of viruses is coordinated with that of their *Leishmania* host. If the presence of viruses exerts a negative effect, whatever small it could be in the mildly infected cells, the latter must be outcompeted by their virus-free counterparts. However, this apparently does not happen, which can be explained by the horizontal transmission of LBVs from the infected to uninfected cells. Secondly, the drastic decrease in the viral load could be explained by massive discharge of viral particles from the cells at the plateau stage. The underlying mechanism likely depends mainly on the host: under the stress conditions (high culture density is likely one of them), *Leishmania* and other trypanosomatids greatly intensify the release of extracellular vesicles [104–106], which may serve as vehicles for viral exit. In this scenario, excessive discharge of viral particles can result in the rise of virus-free cells.

*Leishmania braziliensis* is one of the two most common species (along with *L*. *mexicana*) causing American cutaneous leishmaniasis and the most frequent agent of its hyperergic mucocutaneous form [107]. As the presence of LRV-1 considerably elevates the risk of the development of the latter variant of the disease [22,108,109], a substantial effort has been put to the study of leishmaniaviruses in this and related *Leishmania* spp. belonging to the subgenus *Viannia*. Here, we discovered a novel virus from the family *Leishbuviridae* that has never been detected in the members of this group of trypanosomatids before. The overwhelming majority of studies devoted to viral endosymbionts of *Leishmania* spp. used methods allowing detection of LRVs only [67,110–112]. Therefore, it is not possible to estimate how prevalent LBVs can be in *L*. *braziliensis* and related species, not to say about the impact of such viruses on the clinical symptoms of the disease and other aspects of *Leishmania* biology. By this work we wanted to attract attention of the scientific community to *Lbr*LBV1 as a potential factor of virulence in American leishmaniasis (pending validation *in vivo*) and encourage researchers to test leishmanial strains for the presence of not only LRVs, but also other RNA viruses.

## Supporting information

S1 Fig Maximum likelihood tree of *L*. (*Viannia*) isolates based on maxicircle SNP data. Bootstrap supports (100 replicates) are shown at nodes, but values below 50 are omitted. The scale bar corresponds to the number of substitutions per site. BO17 isolate is in magenta.
(PDF)

S2 Fig PCAdmix ancestry assessment. (A), (B) Scatterplots for PCA-based ancestry estimation (PC1 vs PC2 and PC2 vs PC3, respectively). (C) Local ancestry in bins of 20 SNPs across the whole genome. “+” signs in panels A and B represent a random sample of the parental strains used as control samples for hybrid ancestry estimation. The color scheme in panels (B) and (C) is coordinated with the graphical legend presented in panel (A).
(PDF)

S3 Fig PCAdmix intraspecific ancestry assessment. (A) Scatterplot for PCA-based ancestry estimation (PC1 vs PC2). (B) Local ancestry for selected chromosomes (20 SNPs per bin). The color scheme in panel (B) is coordinated with the graphical legend presented in panel (A).
(PDF)

S4 Fig Secondary structures at the termini of the *Lbr*LBV1 genomic segments. (PDF)

S5 Fig Infection of *Lu*. *migonei* and *Lu*. *longipalpis*. (A), (B) Intensity of infection *in Lu*. *migonei* and *Lu*. *longipalpis*, respectively. Numbers of dissected females are indicated above the bars. * and ** indicate *p-*values below 0.05 and 0.01, respectively. Columns 1–3 and 4–6 in each panel correspond to days 3 and 10 PBM, respectively.
(PDF)

S1 Table SRA accession numbers of *L*. (*Viannia*) isolates used for phylogenetic analyses. (XLSX)

S2 Table SRA accession numbers of *L*. *braziliensis* (no background), *L*. *peruviana* (magenta background), and *L*. *braziliensis–L*. *peruviana* interspecies hybrids (grey background) used for ancestry reconstruction. Samples included into interspecific- and intraspecific hybrid analysis are marked on the right.
(XLSX)

S3 Table Molecular characteristics of the *Lbr*LVB1 genome. (XLSX)
