# Supplementary figures and images for: A novel strain of Leishmania braziliensis harbors not a toti- but a bunyavirus
Source: PLoS Negl Trop Dis. 2024 Dec 27;18(12):e0012767. doi: 10.1371/journal.pntd.0012767 (PMC11717295; doi:10.1371/journal.pntd.0012767)

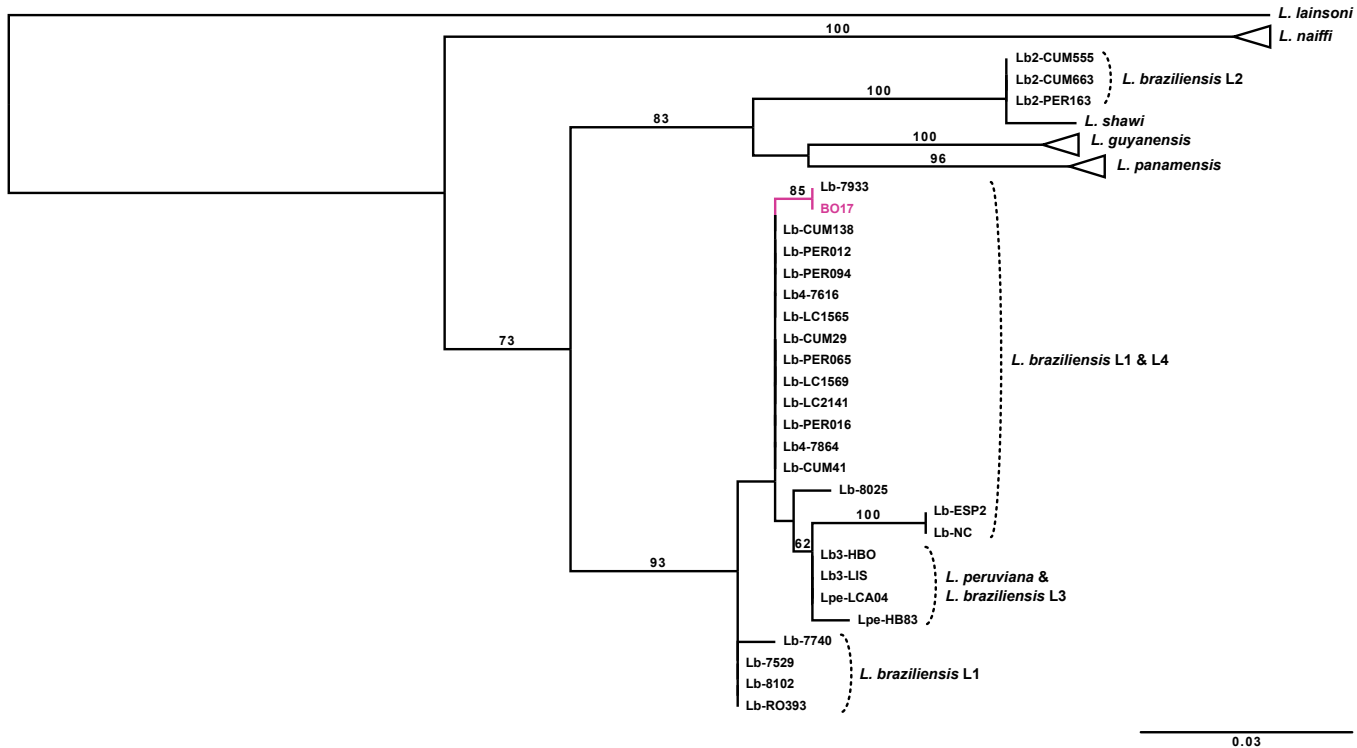

Supplement: S1 Fig — Bootstrap supports (100 replicates) are shown at nodes, but values below 50 are omitted. The scale bar corresponds to the number of substitutions per site. BO17 isolate is in magenta. (PDF) [file pntd.0012767.s001.pdf]

**A**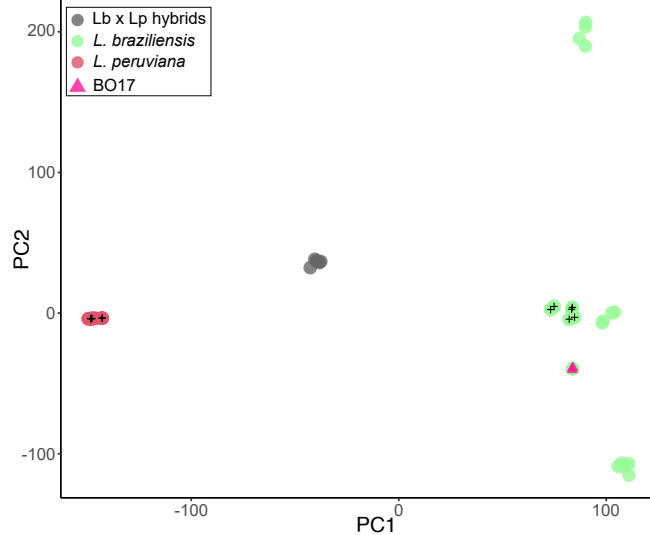**B**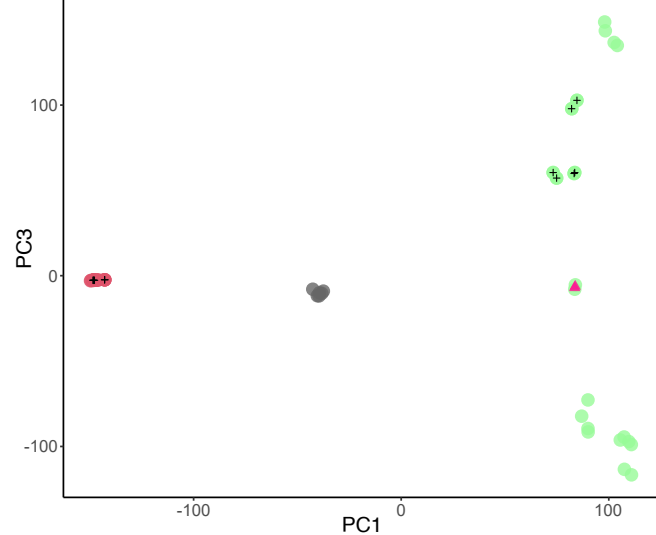**C**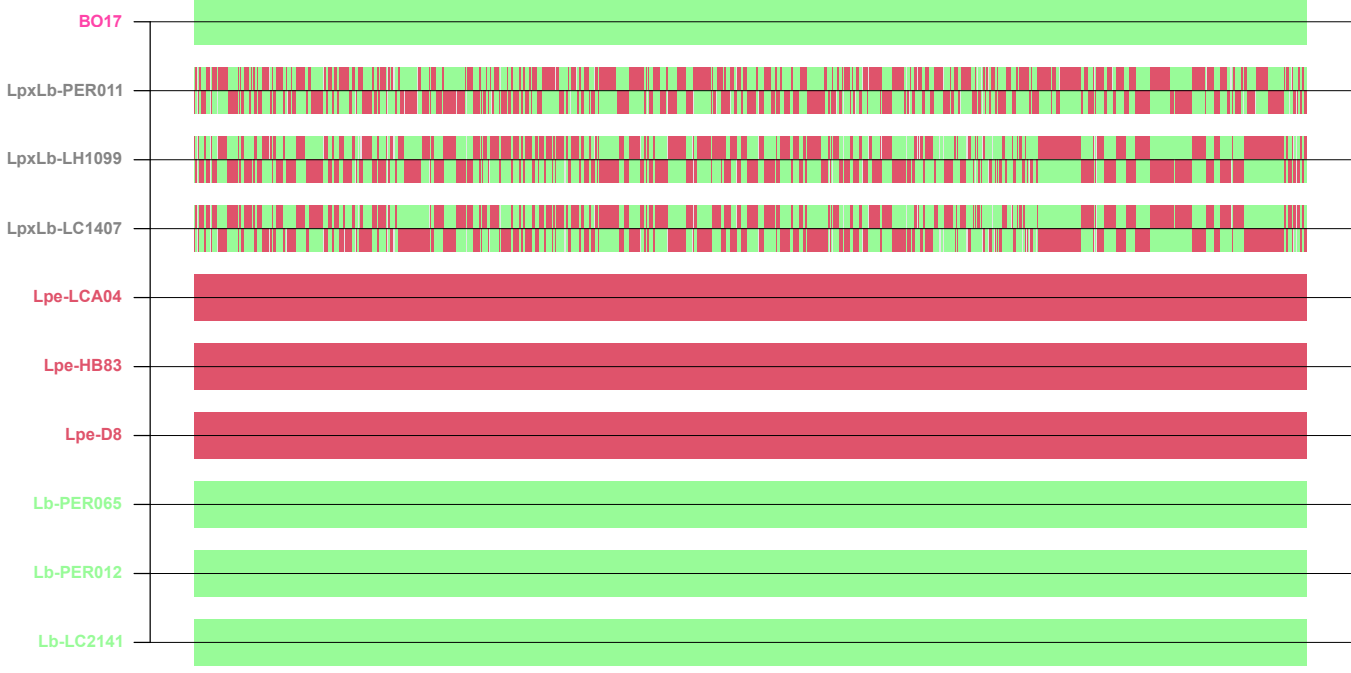

Supplement: S2 Fig — (A), (B) Scatterplots for PCA-based ancestry estimation (PC1 vs PC2 and PC2 vs PC3, respectively). (C) Local ancestry in bins of 20 SNPs across the whole genome. “+” signs in panels A and B represent a random sample of the parental strains used as control samples for hybrid ancestry estimation. The color scheme in panels (B) and (C) is coordinated with the graphical legend presented in panel (A). (PDF) [file pntd.0012767.s002.pdf]

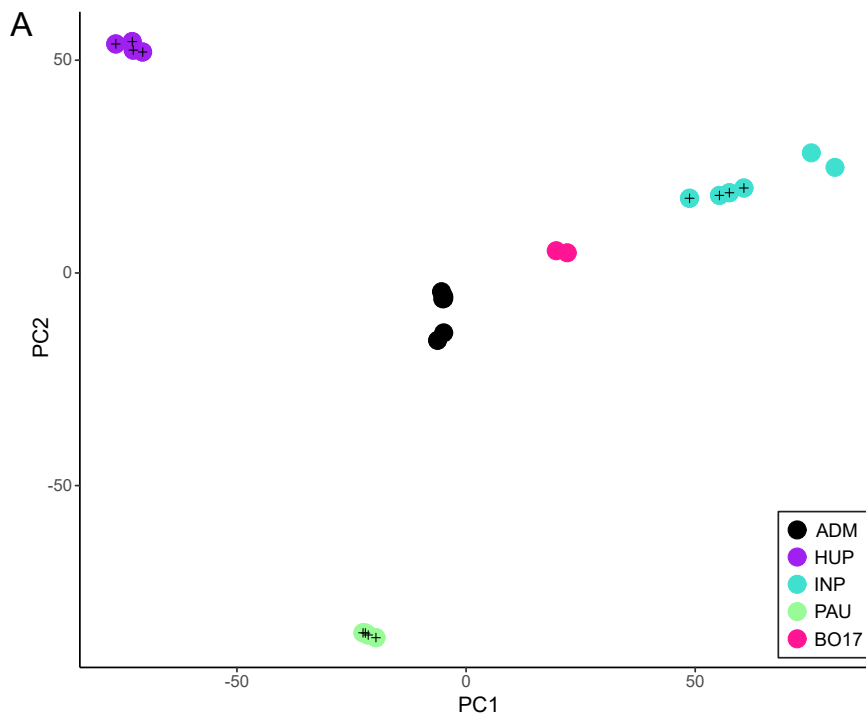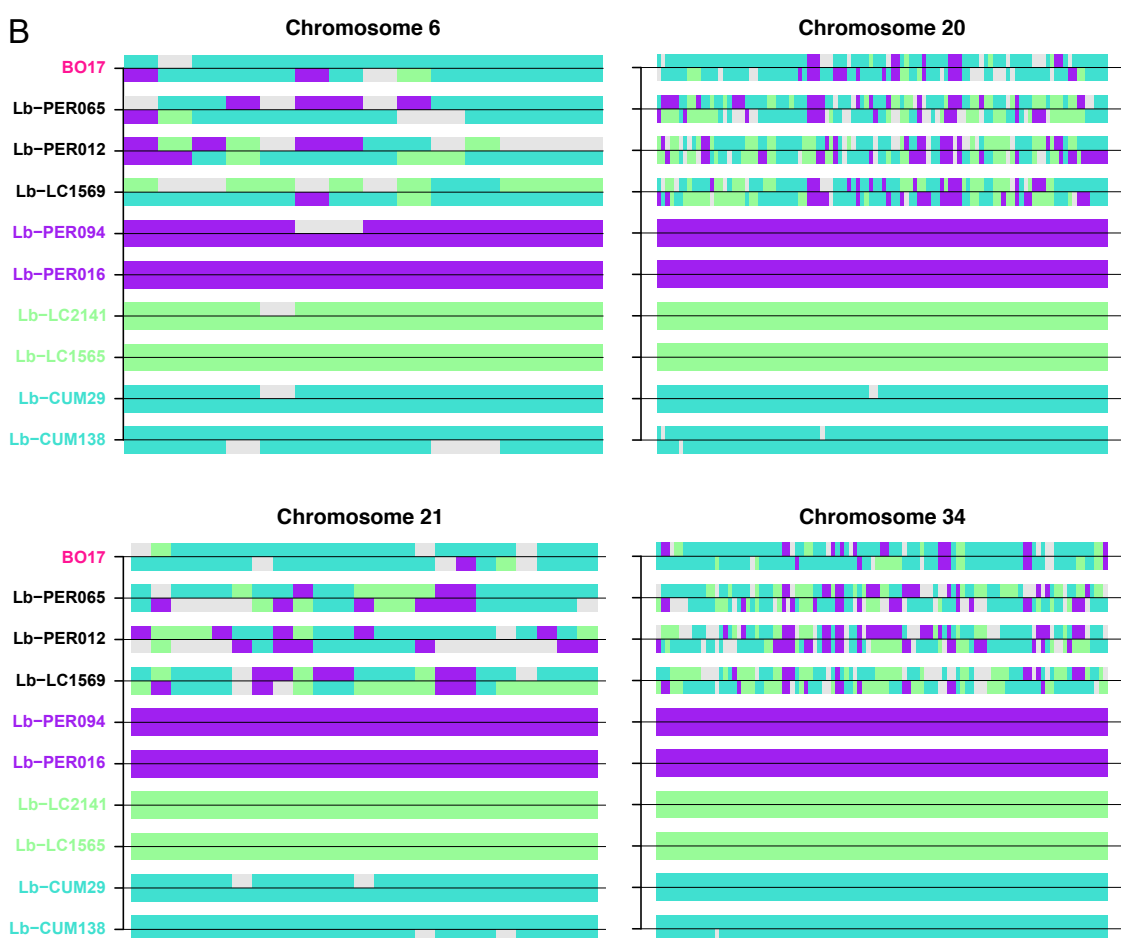

Supplement: S3 Fig — (A) Scatterplot for PCA-based ancestry estimation (PC1 vs PC2). (B) Local ancestry for selected chromosomes (20 SNPs per bin). The color scheme in panel (B) is coordinated with the graphical legend presented in panel (A). (PDF) [file pntd.0012767.s003.pdf]

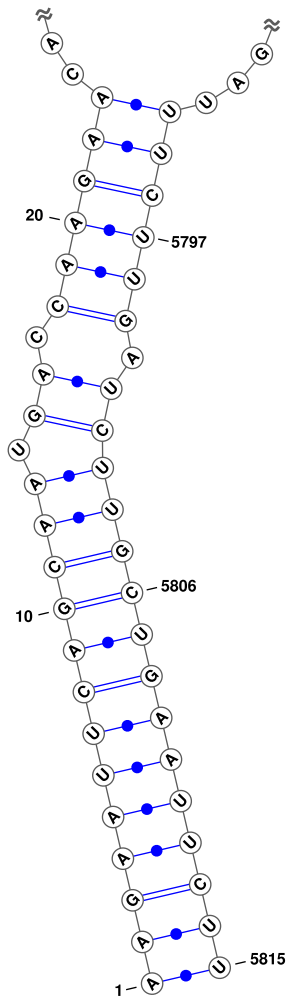

**L**

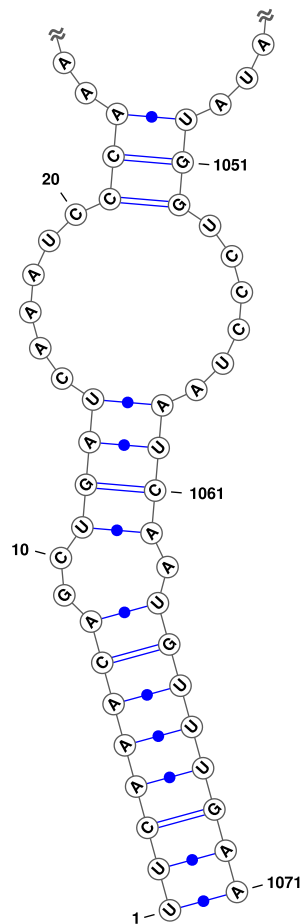

**M**

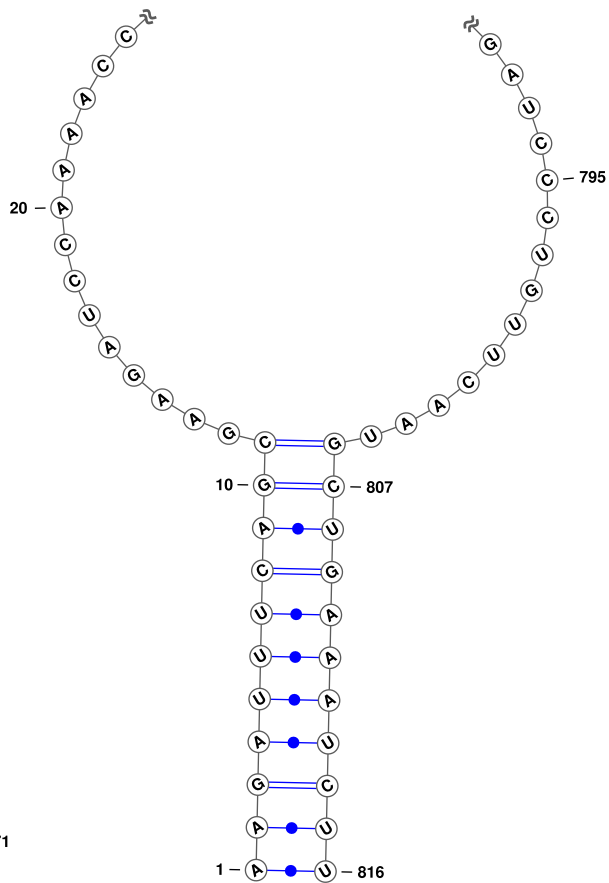

**S**

Supplement: S4 Fig — (PDF) [file pntd.0012767.s004.pdf]

A

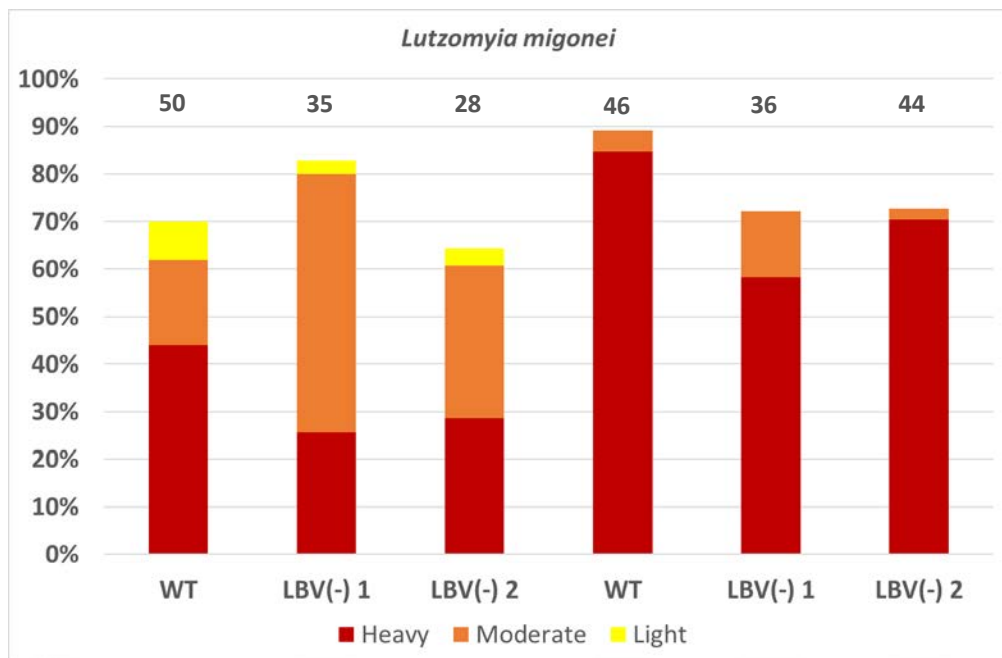

B

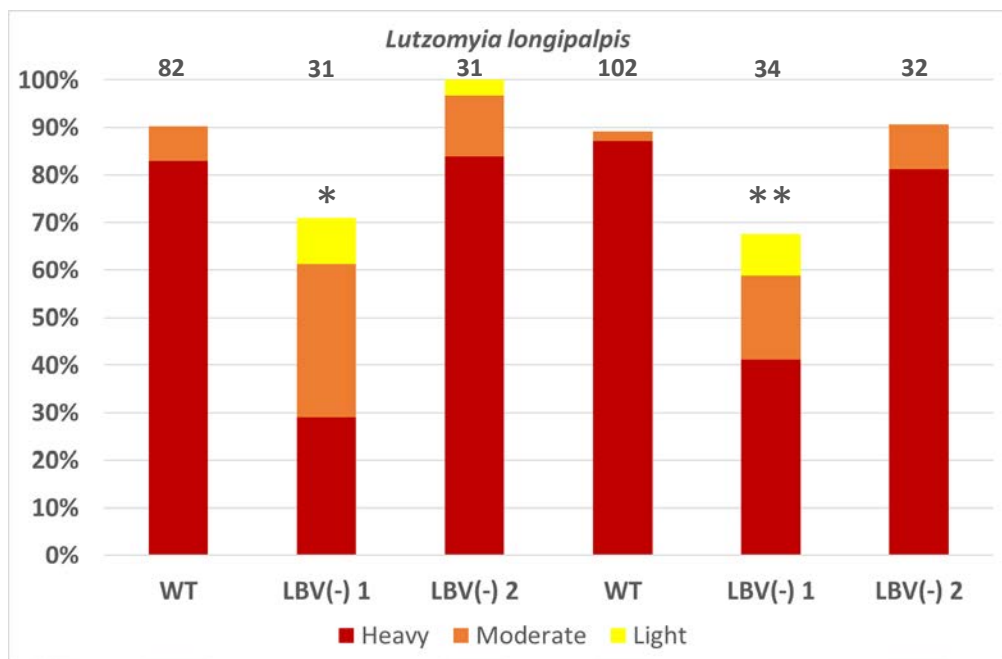

Supplement: S5 Fig — (A), (B) Intensity of infection in Lu. migonei and Lu. longipalpis, respectively. Numbers of dissected females are indicated above the bars. * and ** indicate p-values below 0.05 and 0.01, respectively. Columns 1–3 and 4–6 in each panel correspond to days 3 and 10 PBM, respectively. (PDF) [file pntd.0012767.s005.pdf]
